# Serological diagnostic potential of recombinant outer membrane proteins (rOMPs) from *Brucella melitensis* in mouse model using indirect enzyme-linked immunosorbent assay

**DOI:** 10.1186/s12917-015-0587-2

**Published:** 2015-11-03

**Authors:** Ihsan Muneer Ahmed, Siti Khairani-Bejo, Latiffah Hassan, Abdul Rani Bahaman, Abdul Rahman Omar

**Affiliations:** Department of Veterinary Pathology and Microbiology, Faculty of Veterinary Medicine, Universiti Putra Malaysia, 43400 UPM Serdang, Malaysia; Department of Microbiology, College of Veterinary Medicine, University of Mosul, Mosul, Iraq; Department of Veterinary Laboratory Diagnostics, Faculty of Veterinary Medicine, Universiti Putra Malaysia, 43400 UPM Serdang, Malaysia; Institute of Bioscience, Universiti Putra Malaysia, 43400 UPM, Serdang, Malaysia

**Keywords:** *Brucella melitensis*, rOMPs, FPSR, Mice, ELISA, Recombinant protein

## Abstract

**Background:**

*Brucella melitensis* is the most important pathogenic species of *Brucella spp*. which affects goats and sheep and causes caprine and ovine brucellosis, respectively. Serological tests for diagnosis of brucellosis such as Rose Bengal plate test (RBPT) and enzyme-linked immunosorbent assay (ELISA) usually utilize smooth lipopolysaccharides (S-LPS) as a diagnostic antigen which could give false positive serological reactions. Outer membrane proteins (OMP) of *B. melitensis* have been used as alternative diagnostic antigens rather than S-LPS for differential serological diagnosis of brucellosis, mainly in ELISA with single recombinant OMP (rOMP) as a diagnostic antigen. Nevertheless, the use of single format mainly showed lack of sensitivity against the desired rOMP. Therefore, this study aimed to determine whether a newly developed rOMPs indirect ELISA (rOMPs I-ELISA), based on combination of rOMP25, rOMP28 and rOMP31of *B. melitensis*, has a potential benefit for use in the serodiagnosis of brucellosis.

**Methods:**

In this study, *omp25*, *omp28* and *omp31* of *B. melitensis* were cloned and expressed using prokaryotic pET-32 Ek/LIC system and their respective rOMPs were combined as one coating antigen to develop rOMPs I-ELISA. Three groups of BALB/c mice were used to elicit antibody response. Group 1, infected with *B. melitensis* strain 0331 field strain; group 2, injected with *B. melitensis* Rev.1 vaccine strain and group 3, infected with *Yersinia enterocolitica* O:9. Antibody responses in three groups of mice were investigated using Rose Bengal plate test (RBPT) and rOMPs I-ELISA.

**Results:**

The production of rOMP25, rOMP28 and rOMP31 of *B. melitensis* were achieved and Western immunoblotting analysis demonstrated their reactivity. The RBPT was unable to differentiate the vaccinated mice (group 2) and mice infected with *Y. enterocolitica* O:9 (group 3) and categorized them wrongly as positive for brucellosis. In contrast, the rOMPs I-ELISA was able to differentiate the mice infected with *B. melitensis* strain 0331 (group 1) from both of group 2 and group 3, and recorded 100% sensitivity and 100% specificity.

**Conclusions:**

The results of this study suggested that rOMPs of *B. melitensis* has potential diagnostic ability to differentiate the FPSR in serological diagnosis of brucellosis.

## Background

Brucellosis is one of the most important bacterial zoonoses worldwide [1]. *Brucella melitensis* is the main etiological agent of sheep and goats, and human brucellosis [2]. In control programs of brucellosis, practical solutions for diagnosis of the disease require inexpensive, simple, rapid and specific test to identify the infected animals [3]. Therefore, an indirect diagnosis approach of brucellosis using serological methods mainly Rose Bengal plate test (RBPT), complement fixation test (CFT) and enzyme-linked immunosorbent assay (ELISA) are recommended for large-scale surveillance and/or eradication purposes [4]. These tests usually use S-LPS, part of S-LPS or whole cells as an antigen to detect antibodies to smooth *Brucella* spp. which could give false positive serological reactions (FPSR) results due to difficulties to differentiate between animals vaccinated with *B. melitensis* Rev.1 strain and infected animals [5–7]. Another reason which can lead to FPSR is cross-reactivity with other Gram-negative bacteria like *Yersinia enterocolitica* O:9, *Salmonella* spp. and *Escherichia coli* [2, 8, 9].

The outer membrane proteins (OMP) of *Brucella* spp. were found to be attractive alternative antigens rather than S-LPS for serological diagnosis to minimize the FPSR [10]. *Brucella* OMPs are grouped according to their apparent molecular weights as group 1 (94 or 88 kDa), group 2 (36–38 kDa), and group 3 (25–27 and 31–34 kDa). Group 1 was identified as minor whereas group 2 and 3 OMPs were identified as major OMPs [11]. Group 3 major OMPs have been approved to be useful for the differentiation of antibody responses between naturally infected animals and Rev.1 vaccinated animals [12, 13]. Two genes were identified for the group 3 proteins of *Brucella* and were named *omp25* and *omp31*, coding for 25–27 and 31–34 kDa major OMPs, respectively [14, 15]. In addition, OMP28 of *B. melitensis* has been identified as another member of group 3 OMPs which is coded by *omp28* gene [16]. Others reported that OMP28 is a cytosoluble 28 kDa protein (CP28) which is localized in the periplasm [13], or 26 kDa periplasmic protein (BP26) which is coded by *bp*26 gene [17]. Several individual *omp* genes have been cloned and their expressed proteins were tested in immunoenzymatic assays for serodiagnosis of brucellosis in animals like recombinant OMP25 [18], recombinant OMP28 [19] and recombinant OMP31 [20]. However, lack of sensitivity to detect antibodies against the desired rOMP was the main obstacle facing these recombinant proteins. For that reason, combination of more than one recombinant protein in a single immunoenzymatic test could increase the sensitivity [21].

Small laboratory animals are frequently employed as models in brucellosis research [22]. Among them, BALB/c mice, has been extensively used in brucellosis research for many years mainly due to economic and practical reasons [22–24]. In addition the well-known biology of this murine species, especially the humoral and cellular immunity, makes it the ideal model for brucellosis research [22].

Accordingly, this study aimed to describe the expression and purification of three recombinant proteins, rOMP25, rOMP28 and rOMP31, of *B. melitensis* using *E. coli* expression system. The produced recombinant proteins were combined and used as one coating antigen in an indirect ELISA (I-ELISA) to evaluate its differential serodiagnosis using mouse mode.

## Results

### Construction of pET-32 Ek/LIC-*omp* cloning vector

**Fig. 1:**
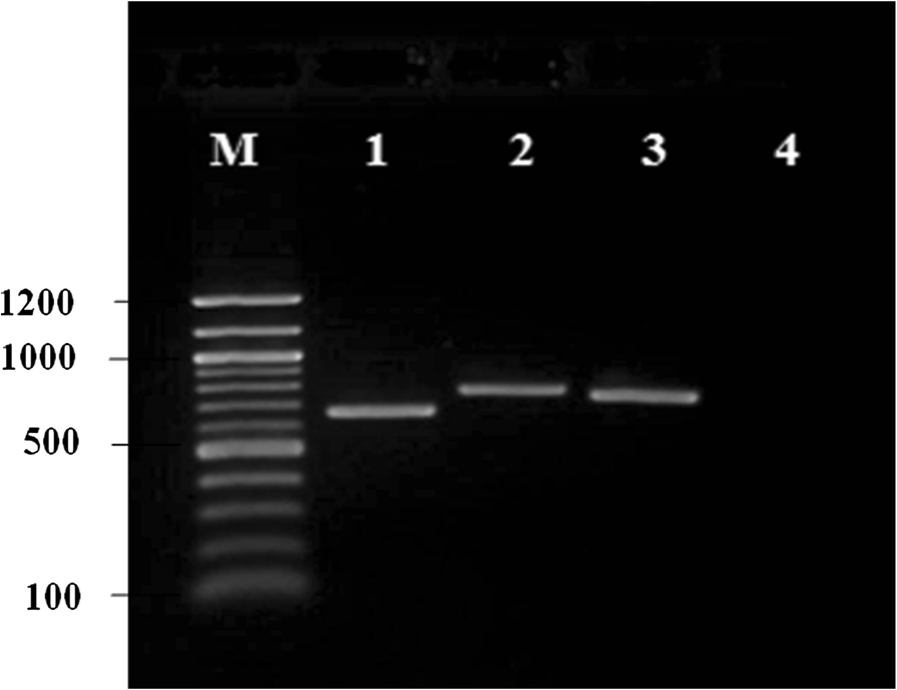
Agarose gel electrophoresis of PCR products of *omp* genes of *B. melitensis* strain 0331. Using gene specific primers that include the indicated 5′ LIC extensions, producing expected bands. Lane M, 100 bp DNA ladder (New England Biolabs, USA); lane 1, *omp25* with product size 668 bp; lane 2, *omp28* with product size 779 bp; lane 3, *omp31* with product size 749 bp; lane 4, negative control

Using polymerase chain reaction (PCR), the *omp*25, *omp*28 and *omp31* gene were amplified from the chromosomal DNA of *B. melitensis* strain 0331 using gene specific primers and produced the expected product sizes of 668, 779 and 749 bp for *omp25*, *omp28* and *omp31* respectively (Fig. 1).

### Nucleotide sequence of *omp25*, *omp28* and *omp31*

Nucleotide sequence analysis of pET-32 Ek/LIC-*omp25,* pET-32 Ek/LIC-*omp28* and pET-32 Ek/LIC-*omp31* inserts revealed the presence of open reading frame (ORF) of 642, 753 and 723 nucleotides for the three genes respectively. The sequences of *omp25*, *omp28* and *omp31* were deposited in the Genbank and assigned the accession numbers [GenBank: JX627633], [GenBank: JX627634] and [GenBank: JX627635] respectively.

### SDS-PAGE analysis and immunoreativity of recombinant fusion proteins

**Fig. 2:**
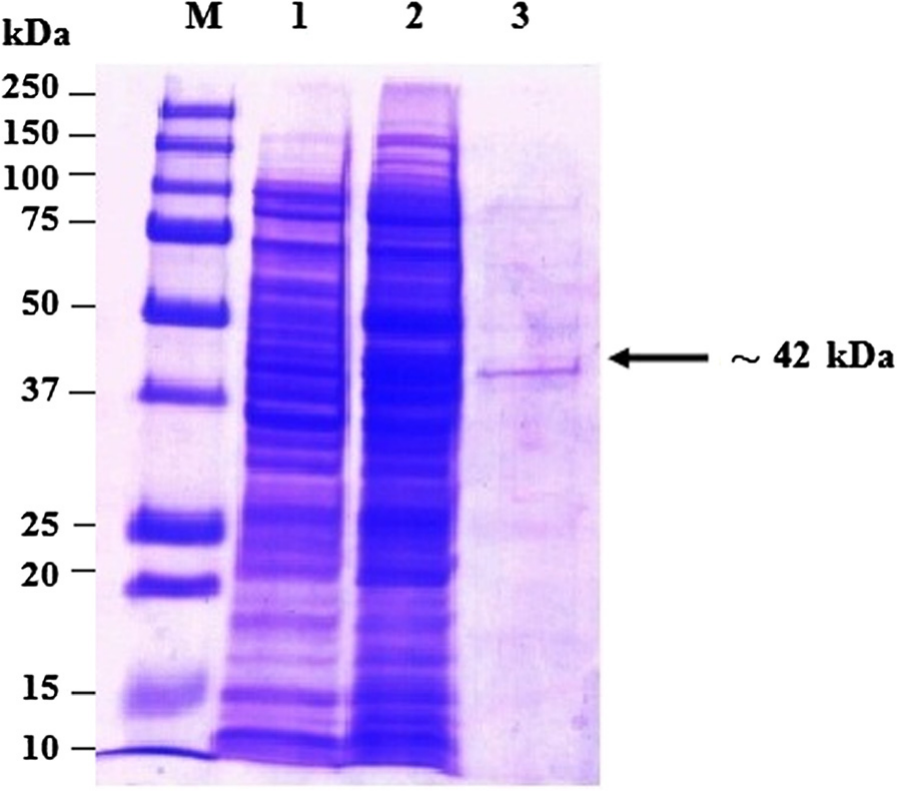
Analysis by SDS–PAGE of rOMP25 fusion protein. Lane M, Protein Marker; lane 1, soluble cell proteins of *E. coli* BL21(DE3) cells with recombinant *omp25* before induction; lane 2, soluble cell proteins of *E. coli* BL21(DE3) cells with recombinant *omp25* after autoinduction; lane 3, rOMP25 fusion protein (*arrow*) purified by affinity purification

**Fig. 3:**
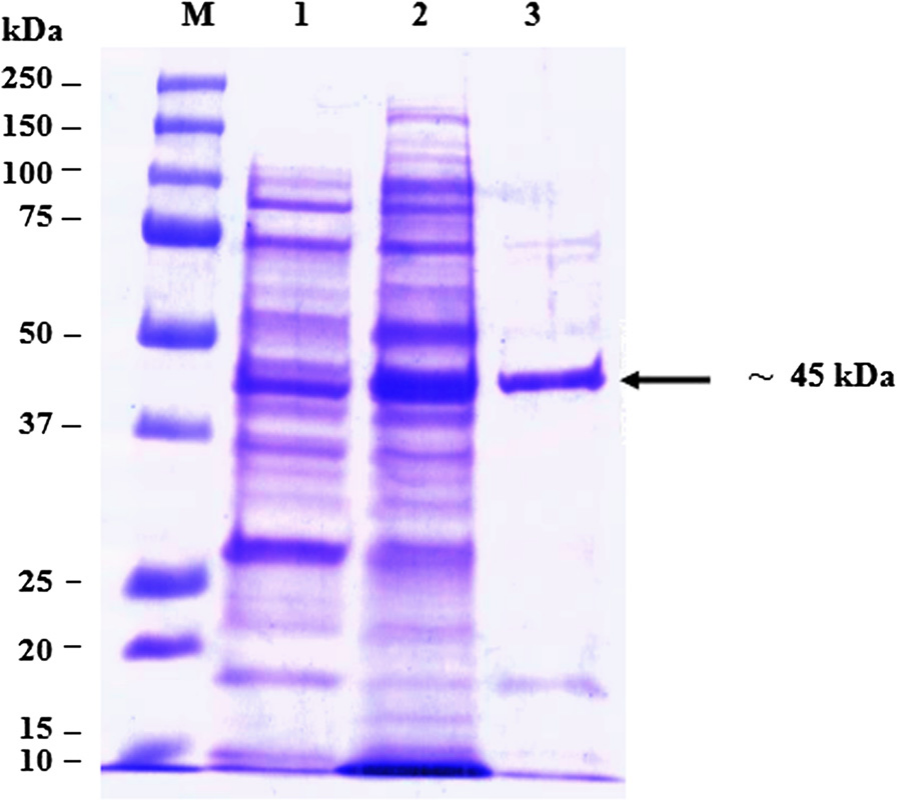
Analysis by SDS–PAGE of rOMP28 fusion protein. Lane M, Protein Marker; lane 1, soluble cell proteins of *E. coli* BL21(DE3) cells with recombinant *omp28* before induction; lane 2, soluble cell proteins of *E. coli* BL21(DE3) cells with recombinant *omp28* after autoinduction; lane 3, rOMP28 fusion protein (*arrow*) purified by affinity purification

**Fig. 4:**
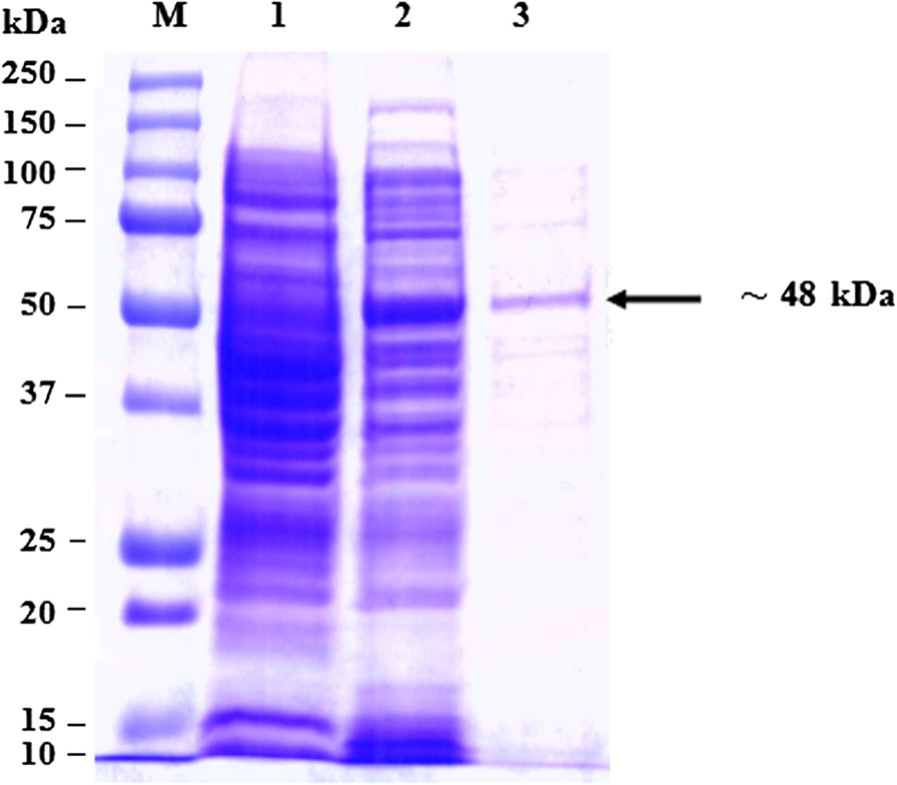
Analysis by SDS–PAGE of rOMP31 fusion protein. Lane M, Protein Marker; lane 1, soluble cell proteins of *E. coli* BL21(DE3) cells with recombinant *omp31* before induction; lane 2, soluble cell proteins of *E. coli* BL21(DE3) cells with recombinant *omp31* after autoinduction; lane 3, rOMP31 fusion protein (*arrow*) purified by affinity purification

**Fig. 5:**
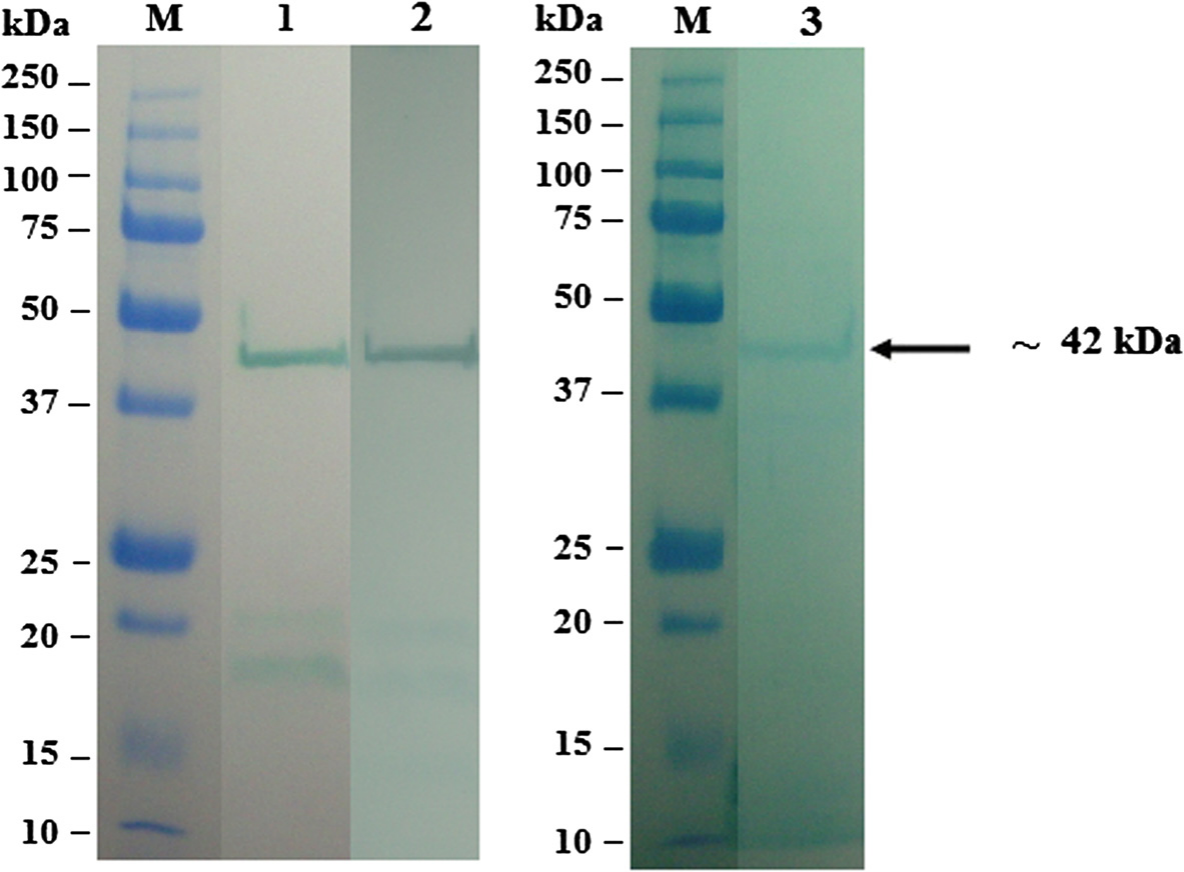
Western immunoblot analysis of purified rOMP25 fusion protein. Lane M, Precision Plus Protein All Blue Standards (Bio-Rad, USA); lane 1, purified rOMP25 detected using His.Tag monoclonal antibody; lane 2, purified rOMP25 detected using S.Tag monoclonal antibody; lane 3, purified rOMP25 detected using rabbit polyclonal antibodies against *B. melitensis* strain 0331

**Fig. 6:**
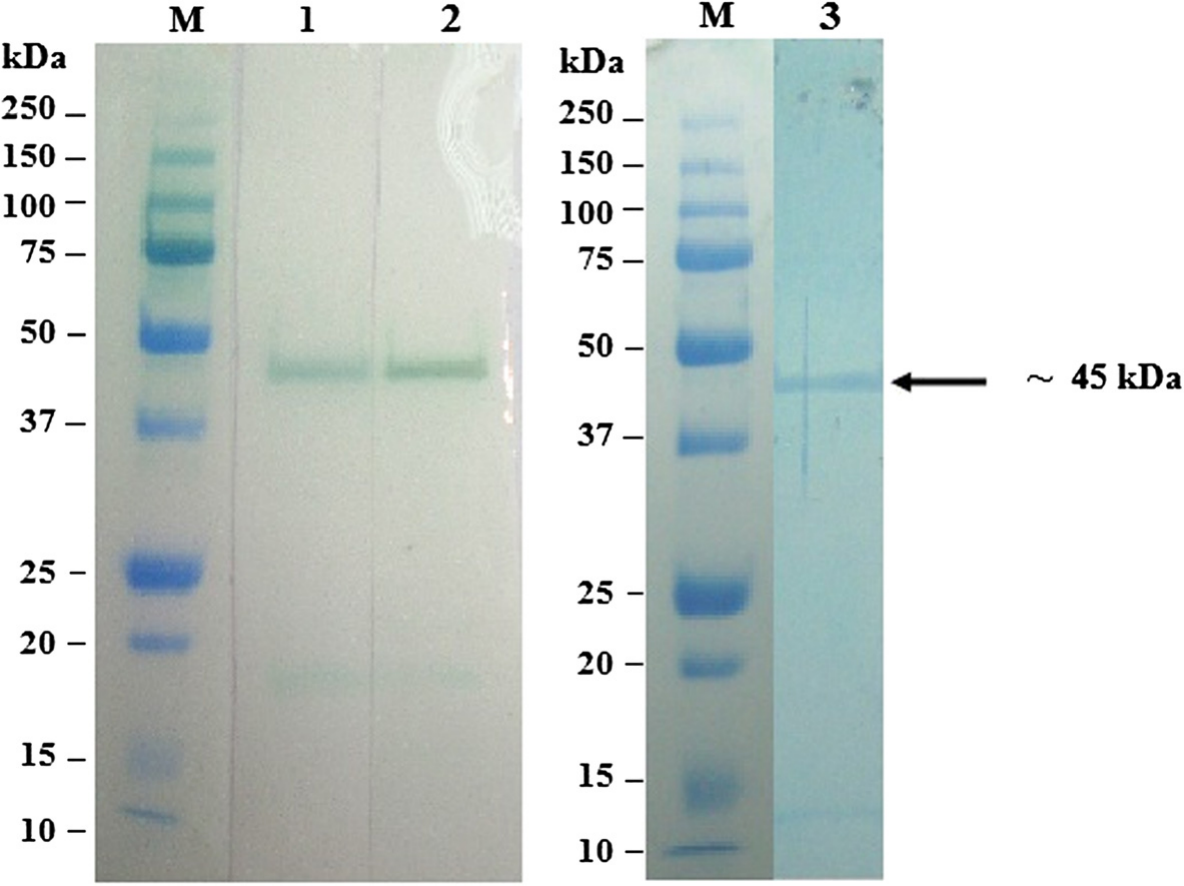
Western immunoblot analysis of purified rOMP28 fusion protein. Lane M, Precision Plus Protein All Blue Standards (Bio-Rad, USA); lane 1, purified rOMP28 detected using His.Tag monoclonal antibody; lane 2, purified rOMP28 detected using S.Tag monoclonal antibody; lane 3, purified rOMP28 detected using rabbit polyclonal antibodies against *B. melitensis* strain 0331

**Fig. 7:**
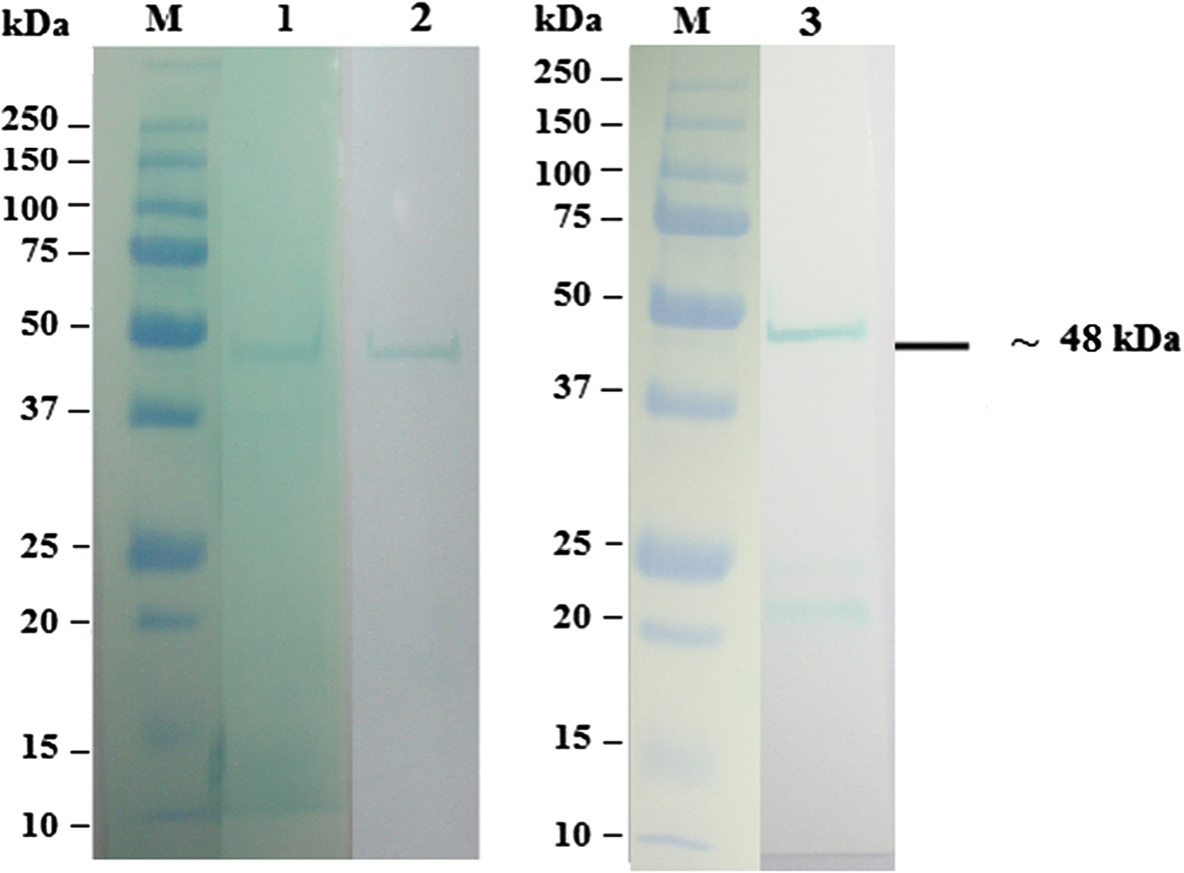
Western immunoblot analysis of purified rOMP31 fusion protein. Lane M, Precision Plus Protein All Blue Standards (Bio-Rad, USA); lane 1, purified rOMP31 detected using His.Tag monoclonal antibody; lane 2, purified rOMP31 detected using S.Tag monoclonal antibody; lane 3, purified rOMP31 detected using rabbit polyclonal antibodies against *B. melitensis* strain 0331

The majority of the expressed rOMP25, OMP28 and OMP31 were found in the soluble fraction. Therefore, the expressed proteins were extracted and purified under native conditions. Approximately final concentration of 50 μg/ml purified recombinant protein could be achieved. The SDS-PAGE analysis showed the presence of the expected 42 k, 45 kDa and Da 48 kDa of purified recombinant fusion proteins (Figs. 2, 3 and 4). The results of Western immunoblot analysis revealed the immunoreactivity of purified rOMP25, OMP28 and OMP31 with the three types of antibodies; two types of monoclonal antibodies (His.Tag and S.Tag HRP conjugated monoclonal antibodies), in addition to rabbit polyclonal antibodies against *B. melitensis* strain 0331 (Figs. 5, 6 and 7).

### Rose Bengal plate test

**Fig. 8:**
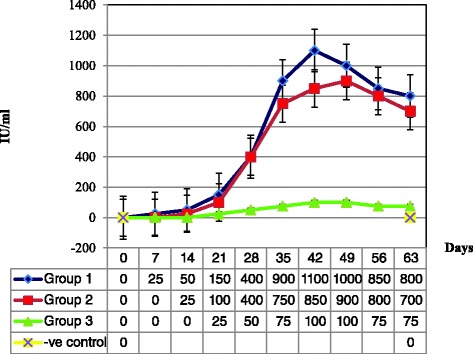
Antibodies titer of mice sera using semi-quantitative RBPT. Group 1, mice infected with *B. melitensis* strain 0331; group 2, mice vaccinated with *B. melitensis* Rev. 1 vaccine strain; group 3, mice infected with *Y. enterocolitica* O:9; (−ve) negative control, mice injected with PBS. ±SE at 95 % CI

The RBPT gave positive results for the tested sera of the three groups of mice, and it was unable to differentiate the antibody response among these groups. However, the RBPT results showed that the mice in group 1 (*n* = 45) were able to elicit high titers of anti-*Brucella* antibodies due to infection with *Brucella melitensis* strain 0331 and peaked 1100 IU/ml at day 42. While, the mice in group 2 (*n* = 45) were able to elicit lower titers of antibodies due to vaccination with *B. melitensis* Rev.1 strain, and peaked 900 IU/ml at day 49. No significant differences (*p* > 0.05) were found between group 1 and group 2 and this due to inability of RBPT to differentiate between the mice vaccinated with *B. melitensis* Rev.1 strain and those infected with *B. melitensis* strain 0331. Although the mice in group 3 (*n* = 45) developed too low titers of antibodies due to infection with *Y. enterocolitica* O:9 (peaked 100 IU/ml at day 42), the RBPT was able to detect these cross reacting antibodies (Fig. 8).

### Recombinant OMPs I-ELISA

**Fig. 9:**
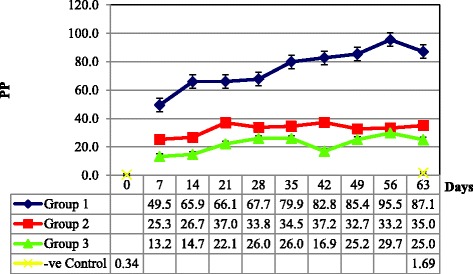
Percentage of positivity (PP) values of the sera from three groups of mice tested using in-house rOMPs I-ELISA. Group 1, mice infected with *B. melitensis strain* 0331; group 2, mice vaccinated with *B. melitensis* Rev. 1 vaccine strain; group 3, mice infected with *Y. enterocolitica* O:9; (−ve) negative control, mice injected with PBS. ±SE at 95 % CI

All mice sera in three groups of mice and negative control were tested by I-ELISA using rOMPs as coating antigen. Using the receiver operating characteristics (ROC), a cut-off value of the positivity percent (PP) ≥ 43.30 was considered positive for brucellosis by rOMPs I-ELISA. The rOMPs I-ELISA results showed that antibodies against rOMPs was elicited as early as day 7 (PP = 49.5) and reach the peak at day 56 (PP = 95.5) in group1. While low antibody titers were detected in both group 2 and group 3 and recorded (PP = 25.3 and 13.2) at day 7, and (PP = 33.2 and 29.7) at day 56, respectively (Fig. 9). Tukey’s test analysis of PP values indicated the presence of significant difference (*p* < 0.05) between group 1 and each of group 2 and group 3. Moreover, by testing sera from mice infected with *B. melitensis* strain 0331, the results of rOMPs I-ELISA recorded 100 % sensitivity (45/45). When sera from mice vaccinated with *B. melitensis* Rev.1 vaccine strain was tested by rOMPs I-ELISA, 100 % specificity (45/45) was recorded. Additionally, 100 % specificity (45/45) was recorded when sera from mice infected with *Y. enterocolitica* O:9 were tested by rOMPs I-ELISA.

## Discussion

In this study, rOMP25, rOMP28 and rOMP31 have been successfully produced and used together to develop rOMPs I-ELISA in a hope to increase the sensitivity of the developed test for differential serodiagnosis of brucellosis using mouse model. The isolation, cloning and expression of *B. melitensis* strain 0331 *omp25, omp28* and *omp31* genes were achieved using prokaryotic system pET-32 Ek/LIC. Using BioEdit sequence alignment editor software, the DNA sequencing results confirm that *omp25, omp28* and *omp31* genes had correct orientation, and they were in the open reading frame (ORF), which in turn enable full amino acids translation of these genes in the subsequent steps. The results of BLASTN alignment showed that *omp25* has 99 % identity with published sequence of *B. melitensis* 16 M reference strain which is due to the difference in nucleotide number 276, which is C instead of T [18, 25]. In order to show whether this nucleotide difference resulting in differences in the amino acids sequence, the alignment of amino acids was performed. The result showed that the nucleotide difference at number 276 in the *omp25* did not cause any differences in the translation of the amino acid glycine (Gly). While, the *omp28* and *omp31* obtained 100 % identity with published sequence of *B. melitensis* 16 M reference strain [16, 26] and [15, 20] respectively. Using amino acid composition feature of BioEdit software, the predicted molecular weight of the tagged fused proteins was estimated to be 17 kDa. In addition, the amino acids of *omp*25, *omp*28 and *omp*31 were also analyzed.

The SDS-PAGE analysis reveals the presence of protein bands of purified rOMP25, rOMP28 and rOMP31 with molecular weight of 42, 45 and 48 kDa respectively. These sizes were agreed with the theoretically molecular weights prediction of the expressed rOMP25, rOMP28 and rOMP31 fusion proteins respectively. Moreover, the results of this study agreed with results obtained by Letesson, et al. [21] and de Wergifosse, et al. [25] for the rOMP25, and Gupta, et al. [27] and Seco-Mediavilla, et al. [28] for the rOMP28, and Vizcaino, et al. [15] and Gupta, et al. [20] for the rOMP31. Furthermore, Western immunoblotting analysis revealed that the rOMP25, rOMP28 and rOMP31 fusion proteins were detected by His.Tag and S.Tag monoclonal antibodies. Additional conformation was made using rabbit polyclonal antibodies against *Brucella melitensis* strain 0331 to assure the immunogenicity of the rOMP25, rOMP28 and rOMP31 fusion proteins, which in turn indicate that these recombinant fusion proteins were expressed in soluble and active form.

The purified rOMP25, rOMP28 and rOMP31 were combined together and named rOMPs. Therefore, mouse model was used to determine the sensitivity and specificity of rOMPs I-ELISA. Accordingly, three groups of mice were set in this study. The first group was infected with *B. melitensis* strain 0331 to demonstrate the antibody response due to infection with *B. melitensis* field strain. While the second group was injected with *B. melitensis* Rev.1 vaccine strain, to demonstrate the antibody response due to vaccination, and the third group was infected with *Y. enterocolitica* O:9, to demonstrate cross reacting antibodies. Antibodies in sera of all mice in the first and second groups were detected using RBPT as early as 15 days after infection, and peaked at 35–49 days, while the sera from mice in the third group showed a much lower antibody reactivity using RBPT. According to OIE, the RBPT is considered as reference test for serological diagnosis of brucellosis. However, the inability to differentiate between infected and vaccinated animals are the main problem facing RBPT because it is based on detection of antibody to S-LPS that is immunodominant in the serological responses of both infected and vaccinated animals [4, 5]. Although low antibodies titer was elicited in group 3, the RBPT was able to detect cross reacting antibodies because of smooth *Brucella* strains share common epitopes on the O-PS with cross-reacting bacteria mainly *Y. enterocolitica* O:9 [2, 9], and in less extent, the natural infection by a number of other Gram negative bacteria can also produce cross reacting antibodies, mainly, *E. coli* O:157 and *Salmonella* spp. [2, 8]. The results of rOMPs I-ELISA, testing sera from three groups of mice, clearly indicated the presence of significant differences between group 1 and each of group 2 and group 3, and this due to ability of rOMPs I-ELISA to detect anti-OMP25, anti-OMP28 or antiOMP31 antibodies in mice infected with *B. melitensis* strain 0331 rather than mice vaccinated with *B. melitensis* Rev. 1 vaccine strain or infected with *Y. enterocolitica* O:9. Earlier studies showed greater accessibility of OMP25 OMP28 and OMP31 epitopes to antibodies generated due to infection with *B. melitensis* [19, 29]. However, previously developed ELISA based on single OMP was challenging due to low sensitivity recorded, like rOMP28 (88.7 %) [19] and rOMP31 (81.8 %) [20]. In our study, the rOMPs I-ELISA recorded 100 % sensitivity (45/45), means that all the mice infected with *B. melitensis* strain 0331 were recognized as positive by our developed ELISA. Additionally, the rOMPs I-ELISA recorded 100 % specificity (45/45), means that all mice vaccinated with Rev. 1 vaccine strain or infected with *Y. enterocolitica* O:9 were recognized as negative.

In summary, in this study, the developed rOMPs I-ELISA was able to increase the sensitivity to detect the infected animals. Moreover, it was also able to differentiate the animals with FPSR. These findings suggesting that rOMPs I-ELISA has potential diagnostic properties which could be used as an effective tool in the differential serodiagnosis of brucellosis.

## Conclusions

Combination of rOMP25, rOMP28 and rOMP31 together clearly increased the sensitivity of the developed rOMPs I-ELISA. In addition, the results also indicated that the rOMPs I-ELISA has the ability to detect specific OMP antibodies due to infection with *B. melitensis* strain 0331 rather than vaccinal or cross reacting antibodies, and this means it has the ability to differentiate between infection with *B. melitensis* and FPSR. However, these result reflect the strict controlled conditions of our study, further evaluations should be performed which involves testing of the developed rOMPs I-ELISA in sheep and goats, as real hosts of *B. melitensis*, to achieve a definitive conclusion about its usefulness in differentiation of FPSR in the serological diagnosis of brucellosis.

## Methods

### Bacterial stain and growth conditions

*Brucella melitensis* strain 0331 Malaysian isolate has been used in this study as a source of the bacterial DNA for cloning and expression of *omp25, omp28* and *omp31* genes. This isolate has been confirmed as *B. melitensis* biovar 1 by Veterinary Laboratory Agency (VLA, Weybridge, UK). Fresh colonies of *B. melitensis* strain 0331 were cultured in Brucella broth (BBL™, BD, USA) for 4 days at 37 °C with continuous shacking; the bacterial cells were harvested by centrifuging.

### PCR amplification and cloning

The DNA extraction and purification were performed following the manufacturer instructions (Qiagen, USA). Whereas, cloning was performed using pET-32 Ek/LIC system (Novagen, USA). Based on published sequence of *B. melitensis* 16 M reference strain [15, 16, 18], specific sets of primers were designed (Table 1) and the *omp25, omp28* and *omp31* genes were amplified using polymerase chain reaction (PCR). The expected products have extra 26 bp, represent vector cohesive overhangs (bold) for efficient cloning with pET-32 Ek/LIC vector. After amplification the produced insert was treated with LIC-qualified T4 DNA Polymerase, and then, annealed to the pET-32 Ek/LIC vector without the need for restriction digestion or ligation. The resultant nicked, circular plasmids are named pET-32 Ek/LIC-*omp25,* pET-32 Ek/LIC-*omp28* and pET-32 Ek/LIC-*omp31* transformed into NovaBlue GigaSingles™ *Escherichia coli* competent cells as initial cloning host. Positive inserts were screened by direct colony PCR using the designed gene specific primers and vector specific primers which includes T7 promoter and T7 terminator. Purified plasmid was sent to First BASE laboratories, (Malaysia) for sequencing. The obtained sequences were analyzed using The Basic Local Alignment Search Tool (BLAST®) of the National Center for Biotechnology Information (NCBI) database and, BioEdit sequence alignment editor software, version 7.0 [30].

**Table 1:** Primers used for amplification of *B. melitensis omp25*, *omp28* and *omp31* genes with vector cohesive overhangs (bold)

No	Primer name	Sequence (5′-3′)	Length	Expected product bp
1.	*B. melitensis omp25* F	**GACGACGACAAG**ATGCGCACTCTTAAGTCTCT	32	668
2.	*B. melitensis omp25* R	**GAGGAGAAGCCCGG**TTAGAACTTGTAGCCGAT	32	
3.	*B. melitensis omp28* F	**GACGACGACAAG**ATGAACACTCGTGCTAGCAATT	34	779
4.	*B. melitensis omp28* R	**GAGGAGAAGCCCGG**TTACTTGATTTCAAAAACGA	34	
5.	*B. melitensis omp31* F	**GACGACGACAAG**ATGAAATCCGTAATTTTGGCGT	34	749
6.	*B. melitensis omp31* R	**GAGGAGAAGCCCGG**TTAGAACTTGTAGTTCAGAC	34	

### Induction of expression and purification of recombinant protein

The positive constructed recombinant clones were used for transformation in expression prokaryotic host *Escherichia coli* BL21(DE3) competent cells (Novagen, USA). Direct colony PCR screening was performed to verify the positive clones using T7 promoter and T7 terminator primer sets. *E. coli* harboring pET-32 Ek/LIC-*omp25,* pET-32 Ek/LIC-*omp28* and pET-32 Ek/LIC-*omp31* plasmids were grown in LB medium and the cells were induced by Overnight Express™ Autoinduction System (Novagen, USA). This system is designed for high-level protein expression with pET expression system without the need to monitor cell growth. To obtain the soluble fraction of the cells, the protein extraction was performed using BugBuster® protein extraction kit with Benzonase® Nuclease (Novagen, USA). Metal chelation chromatography based method was used to purify the rOMP25, rOMP28 and rOMP31 using Ni-NTA His-Bind® Purification Kit (Novagen, USA). The uninduced cell lysate, induced cell lysate and purified rOMPs were analyzed by sodium dodecyl sulfate polyacrylamide gel electrophoresis (SDS-PAGE).

### Production of rabbit hyperimmune serum against *Brucella melitensis* strain 0331

Two six months New Zealand white rabbits were used for raising hyperimmune against *B. melitensis* strain 0331 according to the method of Cloeckaert, et al. [31]. Ethical approval letter, AUP No: 10R1 17/Feb 11-Jan 12 was obtained from Institutional Animal Care and Use Committee (IACUC), Faculty of Veterinary Medicine, Universiti Putra Malaysia, before starting the experiment. The animals were housed under standard conditions at the animal house facility having free access to feed and water *ad libitum*.

The killed antigen was prepared by growing *B. melitensis* 0331 in 100 ml of brucella broth (BBL™, UK) for 3 days at 37 °C using incubator shaker. The broth was pelleted down and the cells were washed in phosphate buffer saline (PBS). Afer that the cells were killed by heating and adjusted to concentration of 10^9^ organism/ml in PBS containing 0.5 % phenol by optical density measurment at 600 nm in a spectrophotometer (eppendorf Biophotometer plus).

The rabbites were injected subcutanously (s.c.) with *B. melitensis* 0331 (killed antigen) 1 × 10^9^ colony forming units (cfu)/ml with Freund’s complete adjuvant (FCA) (Merck, Germany), followed by s.c. injection of two booster doses of *B. melitensis* (killed antigen) 0.5 × 10^9^ cfu/ml with Freund’s incomplete adjuvant (FIA) (Merck, Germany) at days 21 and 35 respectively and the third booster dose was performed at day 45 by s.c. injection of *B. melitensis* (killed antigen) 0.5 × 10^9^ cfu/ml with out any adjuvant. Blood samples were collected from ear vein under sedation at day 0 as self control, to reduce the number of animals included in the study, and subsequently every 2 weeks interval to assess the immune response of the rabbits. The hyperimmune serum against *B. melitensis* was collected by cardiac puncture under anesthesia on day 56. Once the required blood volume was collected, the rabbit was euthanized while still deeply anesthetized. The collected serum was stored at −20 °C until used.

### Western Immunoblotting assay

The expressed rOMP25, rOMP28 and rOMP31 were confirmed to be soluble fusion protein by Western immunoblotting using His.Tag and S.Tag HRP conjugated monoclonal antibodies (Novagen, USA). In addition, rabbit polyclonal antibodies against *Brucella melitensis* strain 0331 was also used to confirm the results. Each purified rOMP was transferred from the unstained polyacrylamide gel onto 0.45 μm nitrocellulose membrane (Bio-Rad, USA) according to the standard procedure by Towbin, et al. [32]. Each blotted membrane which represents one of the rOMP was cut into three membrane parts and blocked using 1 % casein-TBST (Novagen, USA). After washing with TBST, the first membrane part was incubated with a dilution of 1:1500 of His-protein HRP conjugated monoclonal antibody. While the second membrane part was incubated with a dilution of 1:5000 of S-protein HRP conjugated monoclonal antibody. After washing, the colorimetric detection was carried out using TMB peroxidase substrate (Calbiochem, USA) until the color developed. The reaction was stopped by rinsing the membrane in deionized water. The third membrane part was incubated with 1:500 dilution of rabbit hyperimmune serum for 2 h and goat anti rabbit HRP conjugate (KPL, USA) was used as secondary antibody with 1:3000 dilution for 1 h. The remaining washing and colorimetric detection steps were the same as stated for His.Tag and S. Tag.

### Mouse model for evaluation of rOMP25, rOMP28 and rOMP31

Total of (165) five-week-old female BALB/c mice were obtained from the animal facility, Faculty of Veterinary Medicine, Universiti Putra Malaysia (UPM). Ethical approval letter, AUP No: 10R117/Feb 11-Jan 12 was obtained from IACUC, Faculty of Veterinary Medicine, Universiti Putra Malaysia, before starting the experiment. The mice were randomly distributed into three groups of 55 mice and housed in separated plastic cages in controlled animal house facilities for one week to acclimate their environment. The mice were provided with food and water *ad libitum*.

Following the procedure of Jimenez de Bagues, et al. [33], 45 mice in group 1 were infected intraperitoneally (i.p.) with 5 × 10^4^ cfu of *B. melitensis* strain 0331 and 45 mice in group 2 were injected s.c. with *B. melitensis* Rev. 1 vaccine strain 5 × 10^5^ cfu (CZveterinaria, Spain). While in group 3, 45 mice were infected i.p. with *Y. enterocolitica* O:9 3 × 10^4^ cfu [34]. Additionally, for each of the three groups, five mice were sampled pre-infection and five mice were injected with PBS as negative control. Using cardiac puncture technique under general anesthesia, five serum samples were collected from the mice in each group every seven days interval starting at day seven to day 63. The negative controls were sampled at day 63. Once the required blood volume is collected, the mouse was euthanized while still deeply anesthetized using cervical dislocation to produce rapid euthanasia. Sera samples were stored at −20 °C until used.

### Rose Bengal plate test

Sera samples collected from three groups of mice during the experiment period (0–63 days), in addition to negative control samples were tested using semi-quantitative RBPT as described by OIE [35] with some modifications. Briefly, isotonic saline solution was used to prepare serial dilutions of the mice sera (1/2, 1/4, 1/8, 1/16, 1/32, 1/64, 1/128), then equal volume of the diluted seum and RBPT antigen have been mixed and the results was read within 4 min for any positive agglutination. The titer can be calculated by multiplying the dilution factor by the detection limit 25, to give the IU/ml concentration. The RBPT antigen and control sera were obtained from Veterinary Laboratory Agency (VLA, Weybridge, UK).

### Recombinant OMPs I-ELISA

Sera obtained from the three groups of mice were analyzed by rOMPs I-ELISA. The rOMPs coating antigen was produced by combination of equal concentrations of rOMP25, rOMP28 and rOMP31 and used as one coating antigen. The wells of polystyrene plates (Maxisorp, Nunc, Denmark) were coated with 100 μl of purified rOMPs at final concentration of 0.39 μg/ml in 0.05 M bicarbonate buffer (pH 9.6) and incubated overnight at 4 °C. This concentration was determined by checkerboard titration to reach optimal working conditions of sensitivity and specificity following the procedure discribed by Crowther [36]. The wells were emptied and washed three times with phosphate buffer saline-Tween20 (PBST) and then blocked with 5 % skim milk (oxoid, UK). Following incubation 100 μl of mice sera samples, positive and negative control sera diluted 1:200 were added into the plates in duplicate wells and incubated at 37 °C for 1 h. After washing with PBST for three times the plates were incubated with 1:5000 goat anti mouse HRPO IgG (H + L conjugate) (KPL, USA) for 1 h at 37 °C. After washing with PBST, the wells were filled with substrate solution containing TMB (3,3′,5,5′-tetramethyl benzidine) (KPL, USA). Finally, color development was stopped by adding 1 N HCL, after 10 min of incubation of the plates in dark at room temperature. The optical density was measured at 450 nm wavelength using an ELISA reader (Bio-Rad, USA).

### Statistical analysis

IBM SPSS Statistics 19 software (IBM Corp., Armonk, NY, USA) was used for statistical analysis. Differences in antibody titers expressed in IU/ml among the three groups using semi-quantitative RBPT were estimated by comparing the means using one-way ANOVA, and a *P* value < 0.05 was considered statistically significant at 95 % confidence interval (CI). The percentage of positivity (PP) value was calculated as described in the following equation.

$$ \mathrm{P}\mathrm{P}=\frac{\left(\mathrm{test}\ \mathrm{sample}\ \mathrm{O}\mathrm{D}\right) - \left(\mathrm{negative}\ \mathrm{control}\ \mathrm{sera}\ \mathrm{O}\mathrm{D}\right)}{\left(\mathrm{positive}\ \mathrm{control}\ \mathrm{sera}\ \mathrm{O}\mathrm{D}\right) - \left(\mathrm{blank}\ \mathrm{O}\mathrm{D}\right)}\times 100 $$

The PP values were statistically analyzed using on-way ANOVA, Tukey’s multiple comparison test, to detect any significant differences among three groups of mice and a *P* value < 0.05 was considered statistically significant at 95 % CI. The receiver operating characteristics (ROC) was used in order to determine the cut-off value of the trade off between sensitivity and specificity of rOMPs I-ELISA using mice sera from group 1 and 2 including negative controls and the sensitivity and specificity of rOMPs I-ELISA were estimated accordingly [36].

## Abbreviations

RBPT: Rose Bengal plate test
ELISA: Enzyme-linked immunosorbent assay
S-LPS: Smooth lipopolysaccharides
FPSR: False positive serological reactions
OMP: Outer membrane proteins
rOMPs: Recombinant outer membrane proteins
rOMPs I-ELISA: rOMPs indirect ELISA
CFT: Complement fixation test
PCR: Polymerase chain reaction
SDS-PAGE: Sodium dodecyl sulfate polyacrylamide gel electrophoresis
IACUC: Institutional animal care and use committee
PP: Percentage of positivity
CI: Confidence interval
SE: Standard error
